# Role of Transforming Growth Factor-β in Skeletal Muscle Fibrosis: A Review

**DOI:** 10.3390/ijms20102446

**Published:** 2019-05-17

**Authors:** Ahmed Ismaeel, Jeong-Su Kim, Jeffrey S. Kirk, Robert S. Smith, William T. Bohannon, Panagiotis Koutakis

**Affiliations:** 1 Department of Nutrition, Food & Exercise Sciences, Florida State University, Tallahassee, FL 32304, USA; ai18@my.fsu.edu (A.I.); jkim6@fsu.edu (J.-S.K.); 2 Department of Surgery, Capital Regional Medical Center, Tallahassee, FL 32308, USA; jeffrey.kirk@hcahealthcare.com; 3 Department of Surgery, Baylor Scott and White Hospital, Temple, TX 76508, USA; robert.smith@bswhealth.org (R.S.S.); william.bohannon@bswhealth.org (W.T.B.)

**Keywords:** TGF-β, fibrosis, skeletal muscle, myopathy

## Abstract

Transforming growth factor-beta (TGF-β) isoforms are cytokines involved in a variety of cellular processes, including myofiber repair and regulation of connective tissue formation. Activation of the TGF-β pathway contributes to pathologic fibrosis in most organs. Here, we have focused on examining the evidence demonstrating the involvement of TGF-β in the fibrosis of skeletal muscle particularly. The TGF-β pathway plays a role in different skeletal muscle myopathies, and TGF-β signaling is highly induced in these diseases. In this review, we discuss different molecular mechanisms of TGF-β-mediated skeletal muscle fibrosis and highlight different TGF-β-targeted treatments that target these relevant pathways.

## 1. Introduction

Transforming growth factor-beta (TGF-β) is a multifunctional cytokine that includes the isoforms TGF-β 1-3 [1]. The TGF-β signaling pathway is involved in various cellular processes and functions including cell growth, differentiation, and apoptosis [1]. TGF-β also regulates the phenotype and function of fibroblasts and plays a role in myofibroblast transdifferentiation [2,3]. TGF-β functions to promote extracellular matrix (ECM) preservation by enhancing matrix protein synthesis, promoting expression of ECM and profibrotic genes such as type I collagen and connective tissue growth factor (CTGF), and suppressing the activity of ECM degradation proteins such as matrix metalloproteinases (MMPs) [2,3]. Activation of the TGF-β signaling pathway initiates collagen accumulation that is important for wound repair, but certain pathological triggers such as ischemia reperfusion injury (IRI), can lead to enhanced ECM strengthening and progressive fibrosis, which has earned TGF-β the title of “the master regulator of fibrosis” [4,5]. In fact, TGF-β plays a role in the pathogenesis of fibrosis in most organs, including the lung, liver, kidney, heart, and skeletal muscle and is implicated in several diseases and syndromes [6].

Skeletal muscle fibrosis severely impairs muscle function via reduced motile and contractile function. Skeletal muscle fibrosis is characterized by increased area of the ECM in muscle cross-sections, and as a result of enhanced collagen deposition, increased muscle stiffness as well as contracture limits function and mobility [7]. In addition, fibrosis also limits the amount of muscle that is available as a target for therapy and repair. Inability to restore muscle function can lead to disease progression in skeletal muscle myopathies [8].

The purpose of this review is to present the evidence for the involvement of TGF-β in skeletal muscle fibrosis and to explore the molecular mechanisms behind TGF-β’s pro-fibrotic actions in skeletal muscle. We also discuss the role of TGF-β in muscular diseases and discuss potential anti-fibrotic treatments that target the TGF-β pathway. 

## 2. TGF-β Signaling Pathway

The TGF-β superfamily consists of over 50 members that are structurally related, falling into one of three subfamilies: TGF-β, bone morphogenic protein (BMP), and activin [9]. Of all the ligands, TGF-β1 and myostatin are the most implicated in skeletal muscle. Myostatin, a myokine and secreted growth differentiation factor, is a member of the TGF-β superfamily expressed in skeletal muscle. Deletion of myostatin leads to muscle hypertrophy and hyperplasia, which results in an almost doubled muscle mass in mice [10]. TGF-β1 and myostatin have common and contrastive features in their intracellular signaling pathways.

TGF-β1 functions by the active ligand binding to the TGF-β type I receptor (TβRI/ALK-5) or the activin like kinase-1 (ALK-1) receptor and the TGF-β type II receptor (TβRII), which triggers formation of a complex consisting of two TβRI and two TβRII subunits [1]. Following formation of the complex, TβRII subunits phosphorylate TβRI subunits at several serine and threonine residues within the receptor kinase domain, which leads to activation of primarily the SMAD pathway [1]. For myostatin signaling induction, the active myostatin ligand must bind to the activin receptor type IIA (ActR-IIA) or the activin receptor type IIB (ActR-IIB) and either the TβRI/ALK-5 receptor or the activin like kinase-4 (ALK-4) receptor [9]. This also leads to dimerization of the receptors, activation of serine-threonine kinase receptors, and downstream activation of the SMAD pathway. Therefore, both TGF-β1 and myostatin converge at the SMAD pathway. SMAD2 and SMAD3 are the main substrates for TGF-β receptors, referred to as receptor-regulated SMADs, or R-SMADs [11]. Further, SMAD4, also known as Co-SMAD, forms a complex with R-SMADs, and SMAD6 and SMAD7 are inhibitory SMADs (I-SMADs) that can interfere with SMAD interactions with the TGF-β receptors or other SMAD proteins [11].

The nucleocytoplasmic dynamics of these proteins are quite interesting, as SMAD proteins can undergo nuclear translocation without nuclear transport factors, or importins; instead, they can interact directly with nucleoporins [11]. However, in a basal state, R-SMADs (SMAD2/3) are mostly located in the cytosol, likely as a result of cytosolic SMAD-binding factors. Receptor-mediated phosphorylation of the R-SMADs results in a decreased affinity of R-SMADs for the SMAD-binding factors and an increased affinity for nucleoporins [11]. Likewise, SMAD4 shuttles between the cytosol and nucleus in a basal state, but receptor-mediated phosphorylation of the C-terminal of R-SMAD proteins creates a SMAD4 binding site, allowing the R-SMAD/Co-SMAD complex to enter and accumulate in the nucleus, where they act as transcription factors that regulate gene expression [1]. In skeletal muscle, these genes include the myogenic transcription factors, such as the muscle regulatory factors (MRFs) MyoD, Myf5, and myogenin [9]. In addition to this pathway, TGF-β ligands can also function by an alternative pathway involving mitogen-activated protein kinases (MAPKs). However, this pathway is much less well-documented [12].

## 3. TGF-β in Skeletal Muscle Myopathies

### 3.1. Duchenne Muscular Dystrophy

Duchenne muscular dystrophy (DMD) is an X-linked genetic disorder resulting from a mutation of the dystrophin gene that leads to severe muscle degeneration as well as fibrosis of connective tissue [13]. While progress has been made in the understanding of DMD pathology, the underlying mechanisms of fibrosis within dystrophic muscle are still being studied. Several studies have shown that TGF-β1 levels are elevated in DMD patients in both plasma and muscle, and the expression of TGF-β1 is correlated with fibrosis (Table 1) [14,15]. Expression of TGF-β1 and CTGF have also been shown to be upregulated specifically in the muscle cell sarcoplasm and myenteric interstitium of DMD patients compared to controls [16]. The expression levels of TGF-β1 and CTGF are significantly correlated with the degree of pathology and clinical severity of DMD [16]. Furthermore, based on a genome-wide mRNA profiling of DMD and control muscle, the TGF-β pathway is highly induced in patients with DMD compared to controls [17]. TGF-β1 is also upregulated in *mdx* muscle, the mouse model of DMD [18], and blunted TGF-β signaling in *mdx* mice leads to reduced levels of fibrosis [19,20].

### 3.2. Amyotrophic Lateral Sclerosis

Amyotrophic lateral sclerosis (ALS) is a neurodegenerative disorder that leads to loss of motoneurons, causing muscle atrophy and fibrosis [21]. An earlier study assessing TGF-β1 concentrations in serum and cerebrospinal fluid (CSF) of ALS patients found that TGF-β1 serum levels were significantly higher in terminal clinical status ALS patients than controls, and CSF concentrations of TGF-β1 were significantly positively correlated with the duration of ALS [22]. In muscle samples, TGF-β1 concentrations have also been shown to be increased compared to controls and to correlate with muscle strength [23]. Likewise, in G93A SOD1 mice, the mouse model of ALS, TGF-β1 increases with increased disease progression, implicating TGF-β1 as a biomarker of ALS in skeletal muscle [23]. Extensive fibrosis has been documented in skeletal muscle of G93A SOD1 mice, as these mice exhibit an increase in ECM components and increased amounts of the fibrotic proteins, fibronectin and collagen type I, at the perimysium and endomysium compared to control mice. This enhanced fibrosis is accompanied by an induction of TGF-β signaling, assessed by levels of phosphorylated, active SMAD3 [24]. Expression levels of TGF-β1 negatively correlate with lifespan in G93A SOD1 mice as well, and inhibition of TGF-β signaling extends survival [25].

### 3.3. Peripheral Artery Disease

Peripheral artery disease (PAD) is an atherosclerotic disease presenting as narrowing of the arteries of the lower extremities [26]. Notably, IRI is a main feature of PAD, as the lower extremity muscles of patients are under ischemia during activity, but experience reperfusion and re-oxygenation during rest. Constant effort-induced cycles of ischemia and reperfusion result in increased reactive oxygen species (ROS) formation and muscle oxidative damage, which leads to a myopathy in the affected leg muscles of patients that is characterized by myofiber degeneration and increased fibrosis [27].

Expression of TGF-β1 in PAD gastrocnemius muscle has been shown to be almost three times higher than controls [28]. Collagen deposition is also increased in PAD patients compared to controls and increases with advancing disease stage [28]. TGF-β1 expression also progressively increases with advancing disease stage and positively correlates with collagen density. Finally, TGF-β1 expression is associated with fibroblast accumulation in areas of collagen deposition, further supporting the idea of a TGF-β-induced progressive fibrosis in PAD skeletal muscle [28].

### 3.4. Marfan Syndrome

Marfan syndrome (MFS) is an inherited disorder that affects connective tissue, caused by mutations in *FBN1*, the gene encoding the ECM protein fibrillin-1 [29]. MFS is also associated with a severe myopathy, characterized by significant decreases in muscle mass, an inability to increase muscle mass, and an increase in skeletal muscle fibrosis [29]. Notably, circulating TGF-β1 concentrations have been shown to be elevated in patients with MFS compared with healthy individuals, and this has also been demonstrated in *FBN1* mutant mice [30].

### 3.5. Aging-Associated Fibrosis

TGF-β1 is believed to also play a role in the muscle impairment and fibrosis that accompanies the aging process. During normal aging, muscle cells increase TGF-β1 levels, and transition to a more fibrotic phenotype [31]. Skeletal muscle gene expression of TGF-β1 has been shown to be higher in older versus younger adults [32]. Results of a global gene expression profiling suggested that aging muscle demonstrates an increase in expression for genes coding for TGF-β1 [33]. This phenomenon is believed to be due to one of two factors. First, the increased TGF-β1 expression may be a result of age-associated chronic inflammation, which drives fibroblast activation [33]. Second, this may reflect an attempt to repair accumulated tissue damage [33]. 

### 3.6. Other Myopathies

Increased TGF-β signaling has also been linked to several other acquired myopathies. For example, muscle atrophy induced by several conditions including hypoxia, microgravity, disuse, and cancer cachexia have all been associated with increased TGF-β1 and/or myostatin expression and activation [34,35,36,37]. Alterations in TGF-β signaling are also thought to be one of the molecular mechanisms that underlie sarcopenia, the age-related loss of skeletal muscle mass and function, due to the negative regulation of skeletal muscle development induced by TGF-β1 and myostatin [38]. Likewise, immobilization and injury, which are associated with acute muscle wasting, weakness, and muscle fibrosis, also show strong inductions of TGF-β [38]. For example, atrophic myofibers from patients with acute quadriplegic myopathy show increased stimulation of the TGF-β pathway [39]. Similarly, there is a significant increase in muscle fibrosis that contributes to muscle stiffness following many muscle injury models, such as rotator cuff tears. Interestingly, in a rat model for rotator cuff tears, it was shown that the significant increase in fibrosis in the rotator cuff muscle was associated with a concomitant increase in TGF-β1 gene and protein expression, further emphasizing the role of TGF-β in skeletal muscle pathology and impaired regeneration [40].

## 4. TGF-β-Induced Muscle Fibrosis: In-vitro and in-vivo Evidence

The earliest evidence demonstrating the involvement of TGF-β1 in skeletal muscle fibrosis comes from an in-vitro study by Li et al. [41]. Specifically, the C2C12 mouse myoblast cell line was cultured with varying concentrations of TGF-β1. Expression of myogenic proteins including desmin, MyoD, and myogenin decreased significantly after TGF-β1 treatment compared to non-treated cells [41]. In contrast, non-treated cells expressed low levels of fibrotic proteins including α-smooth muscle actin (α-SMA), fibronectin, and vimentin, and treatment with TGF-β1 led to up-regulated fibrotic protein expression [41]. 

Similar results have also been reported in-vivo. In a study by Mendias et al., mice treated with recombinant TGF-β1 displayed increased collagen I content of extensor digitorum longus (EDL) muscle ECM, increased procollagen Iα2 expression of the tibialis anterior (TA) muscle, and enhanced ECM accumulation compared to vehicle-treated mice [42]. The morphological changes in these mice were also accompanied by reduced contractile forces, as the maximum isometric force production of the EDL muscle was dramatically reduced in TGF-β1-treated mice [42]. In fact, compared to control muscle, TGF-β1-treated muscle showed a 75% reduction in maximum twitch force, a 66% reduction in specific twitch fore (normalized by cross-sectional area (CSA)), and an 89% increase in half-relaxation time [42]. Notably, this study indicated that TGF-β1 can directly induce muscle fibrosis and reductions in force-generating capacity independent of muscle injury or disease. In addition to fibrosis, TGF-β1-treated mice also exhibited significant muscle atrophy, indicated as reductions in muscle CSA of up to 38%. However, due to the extensive accumulation of collagen, there were no observed changes in whole muscle mass [42]. 

A study published a year later by Narola et al. suggested a dose-dependent response to TGF-β1 [43]. In the study, a tet-repressible muscle specific TGF-β1 mouse model (*TRE-TGF-β1/mCK-tTA*) was used, with *TGF-β1* transgene expression induced by discontinuation of doxycycline [43]. The onset of disease phenotype, assessed as loss in body weight with concomitant muscle weakness (measured by grip strength), greatly differed among the mice. Out of 20 *TRE-TGF-β1/mCK-tTA* mice, 40% displayed disease phenotype within 2 weeks and were categorized as early onset (EO), and the remaining 60% were categorized as late onset (LO) (of which 30% displayed disease phenotype at 5-12 weeks and 30% did not show disease phenotype in the entire 15-week study period) [43]. The TGF-β1 protein expression in the skeletal muscle of LO mice was only 4 times greater than control mice, but there was a 234-fold elevation of TGF-β1 in the EO mice. Histochemical analysis of the quadricep muscles demonstrated that the mice over-expressing TGF-β1 (both EO and LO) displayed excessive collagen deposition, with collagen accumulation reaching almost 8-fold higher in the EO group than the control mice, and approximately 2-fold higher in the LO group compared to the control group. Myofiber size was also significantly reduced in both the EO and LO groups compared to controls. In addition, hindlimb muscle strength was significantly reduced in the EO group by 11.2% compared to the control mice [43].

## 5. Mechanisms

TGF-β is produced by different cell types, including T cells, macrophages, fibroblasts, and epithelial cells [44]. The TGF-β pathway can then lead to the activation of different cell lines, including resident fibroblasts, adult muscle-derived stem cells, fibrocytes, nerve-associated cells, and inflammatory or perivascular cells, into myofibroblasts, which are initiators of the fibrotic process [44]. Additionally, the recent discovery of an adult muscle progenitor population, the fibro-adipogenic progenitors (FAPs) has provided new insight into the cellular mechanisms of skeletal muscle fibrosis. Specifically, FAPs do not differentiate into muscle fibers, and rather only differentiate into adipocytes or myofibroblasts depending on the local environment [45]. Macrophages play an important role in this fate determination, as macrophages are associated with fibrosis in human DMD muscle as well as *mdx* mouse muscle [46,47].

Following acute injury, for example, there is a large increase in FAPs to support muscle repair. Under normal conditions however, the FAP populations are returned to basal levels by apoptosis to prevent differentiation into fibroblasts which could increase fibrosis [48]. This apoptosis was shown by Lemos et al. to be induced by tumor necrosis alpha (TNF) that is expressed by pro-inflammatory macrophages [48]. Interestingly, they also showed that expression of TGF-β by anti-inflammatory macrophages blocks this TNF-driven apoptosis of FAPs and increases collagen expression and fibrosis. More recently, Juban et al. also showed that pro-inflammatory macrophages can also produce latent TGF-β1, which is activated by enzymes provided by FAPs, ultimately leading to collagen production by fibroblasts in DMD [47]. It is also important to note that FAP cells are not confined as source of fibrosis in dystrophic muscle only. For example, Davies et al. demonstrated that in a mouse model of rotator cuff tear, TGF-β promoted muscle fibrosis by preventing FAP apoptosis as well [49]. Together, these studies point to the complicated cross-talk between both pro- and anti-inflammatory macrophages and FAPs that leads to increased fibrosis in primary skeletal muscle diseases as well as following conditions such as muscle injury.

### 5.1. Transcription Factors

**Figure 1:**
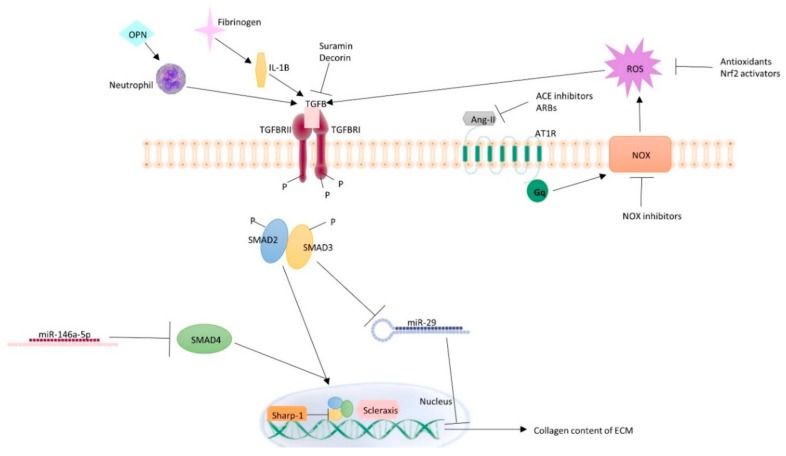
Mechanisms of TGF-β-mediated skeletal muscle fibrosis. Transforming growth factor beta (TGFB) promotes extracellular matrix (ECM) preservation by promoting the expression of profibrotic genes such as type I collagen. Members of the TGFB superfamily function by binding to TGFB type I receptor (TGFBRI) and TGFB type II receptor (TGFBRII). This triggers formation of a complex and phosphorylation and activation of SMAD2 and SMAD3 proteins, which then form a complex with SMAD4, allowing SMADs to be transported to the nucleus to bind transcription factors such as scleraxis. Another transcription factor, Sharp-1, associates with SMAD3 and antagonizes TGFB signaling. microRNA 29 (miR-29) leads to downregulated expression of pro-fibrotic genes. miR146a-5p leads to downregulated expression of SMAD4. Fibrinogen and osteopontin (OPN) are inflammatory molecules that lead to increased TGFB expression through increased neutrophil count and interleukin 1 beta (IL-1β) expression, respectively. Angiotensin II (Ang-II) signals through the angiotensin II receptor type I (AT-1R), coupled to heterotrimeric Gq proteins, stimulating NADPH oxidase (NOX)-derived reactive oxygen species (ROS) formation. Enhanced oxidative stress is another mechanism that leads to enhanced TGFB expression and signaling. Therapeutics targeting this pathway include antioxidants, NOX inhibitors, NF-E2-related factor 2 (Nrf2) activators, angiotensin-converting enzyme (ACE) inhibitors, and angiotensin receptor blockers (ARBs). Suramin is a TGFB receptor antagonist and decorin is an inhibitor or TGFB signaling that have also been implicated as TGFB-targeted treatments of skeletal muscle fibrosis.

Recent data has also provided new insight into the molecular mechanisms behind TGF-β-mediated muscle fibrosis (Figure 1). For example, studies have suggested that TGF-β may regulate fibrosis by modifying the expression of different transcription factors. One such transcription factor is scleraxis, which is expressed in fibroblasts and drives proliferation and type I collagen synthesis [50]. In a study by Mendias et al., treatment of mice with TGF-β led to increased levels of scleraxis and a concomitant increase in the activation of fibroblasts as well as collagen I content of the ECM in EDL muscles, suggesting that one way by which TGF-β may regulate fibrosis is by modifying scleraxis expression [42].

Furthermore, another transcription factor that has been implicated in muscle regeneration is Sharp-1. Following freeze injury, mice lacking Sharp-1 (Sharp-1^−/−^) demonstrated enhanced quadriceps muscle fibrosis and an increase in the number of cells positive for α-SMA compared to WT mice [51]. Sharp-1^−/−^ mice also demonstrated sustained elevations of TGF-β expression and signaling (phosphorylated SMAD2 and SMAD3) [51].

### 5.2. Inflammation

Fibrinogen, an acute phase protein that functions primarily to occlude blood vessels, also plays a role in tissue fibroblast proliferation [52]. Fibrinogen accumulation and deposits as well as TGF-β expression were shown to be increased in *mdx* mouse muscle [53]. Further, fibrinogen depletion reduced the expression of TGF-β and collagen I and attenuated fibrosis [53]. Fibrinogen-deficient mice also exhibited a reduction in the number of macrophages. In contrast, fibrinogen stimulation led to enhanced TGF-β expression by macrophages; however, this effect was lost when cells were treated with an interleukin 1 beta (IL-1β) neutralizing antibody. Together, these data suggest that fibrinogen may cause an inflammatory macrophage response resulting in the synthesis of IL-1β, which leads to increased TGF-β expression by the macrophages [53].

Another inflammatory molecule that may play a role in muscular dystrophy-mediated fibrosis is osteopontin (OPN), a matricellular glycoprotein which functions as a cytokine and promotes migration and survival of immune cells [54]. Vetrone et al. first found that OPN is elevated in both the serum of *mdx* mice and muscle tissue of DMD patients [55]. They further studied the effect of OPN deletion in *mdx* mice, generating OPN-null, dystrophin-deficient, double-mutant mice (DMM). OPN deletion led to a reduction in neutrophils and natural killer T (NKT)-like cells and an increase in regulatory T cells (Tregs) in DMM muscles compared to muscles of *mdx* mice expressing OPN. These changes in inflammatory cells also were accompanied by a reduction in TGF-β, which correlated with increased strength and reduced fibrosis [55]. This suggests that OPN may lead to an inflammatory cascade that results in greater intramuscular TGF-β signaling, promoting fibrosis.

### 5.3. Oxidative Stress

Angiotensin II (Ang-II) is implicated in skeletal muscle fibrotic disorders as well. Ang-II induces ECM proteins including type III collagen and fibronectin via signaling through the angiotensin II receptor type I (AT-1R) [56]. Incubation of C2C12 cells with Ang-II led to an increase in TGF-β1 and CTGF expression, but pre-incubation with the AT-1R blocker losartan abolished this effect [57]. Additionally, apocynin, an inhibitor of NADPH oxidase (NOX) activity, as well as the antioxidant N-acetyl cysteine (NAC) also prevented Ang-II-induced expression of TGF-β1 and CTGF, suggesting that Ang-II-induced expression of the pro-fibrotic factors involves NOX-induced ROS [57]. This is consistent with the results of another study in which inhibition of ROS production by NOX inhibitors led to decreased Ang-II dependent expression of the ECM proteins collagen-III and fibronectin as well as CTGF levels [58].

Enhanced oxidative stress from the endoplasmic reticulum (ER) is another pathological mechanism associated with myopathies and a fibrotic muscle phenotype. Pozzer et al. showed that in a mouse model of ER hyper-oxidation (over-expression of ER oxidoreductin 1 [ERO1], the main protein disulfide oxidase of the ER that generates hydrogen peroxide and knockout out selenoprotein N (SEPN1), an ER reductase), there was an up-regulation of the TGF-β pathway and TGF-β target genes, and a reduction in muscle tension [59]. In vitro, SEPN1 knock-down myoblasts also showed higher levels of phosphorylated (active) SMAD2 and SMAD3, as well as high levels of procollagen I, which was worsened by over-expression of ERO1 [59]. Decreased tissue ascorbic acid (antioxidant) levels were associated with enhanced TGF-β signaling in vivo, and ascorbic acid supplementation normalized TGF-β activity in SEPN1 knock-down myoblasts in vitro. Further, mice lacking L-Gulonolactone oxidase (Gulo KO mice), an enzyme required for ascorbic acid synthesis, showed a fibrotic phenotype in the gastrocnemius muscle, reduced muscle strength, and hyperactivity of the TGF-β pathway, which was attenuated with increasing doses of ascorbic acid treatment [59]. 

### 5.4. MicroRNAs

Finally, there is evidence that microRNAs (miRs) are also involved in TGF-β signaling. miRs are non-coding RNAs ~22 nucleotides long that suppress translation of target genes by binding to the 3’-untranslated regions (3’-UTRs) or by degrading target gene mRNAs [60]. Recently, miRs have been shown to play a role in myogenic regulation as powerful regulators of post-transcriptional gene regulation. For example, miR-29, which has been established as anti-fibrotic in pulmonary fibrosis [61], may also play a similar role in skeletal muscle. Notably, miR-29 levels are significantly reduced in muscle of patients with DMD and muscles of *mdx* mice [62,63]. Additionally, in-vitro, over-expression of miR-29 in C2C12 cells was shown to inhibit fibrogenic differentiation by suppressing the expression of Collagen 1A1, 1A2, and 3A1, while knock-down of miR-29 had the opposite effect [64]. Further, miR-29 has been shown to be under negative regulation by TGF-β, as treatment of C2C12 cells with TGF-β led to reduced expression and activity of miR-29, and reversed the miR-29-induced suppression on Collagens and α-SMA [64]. This finding suggests that miR-29 upregulation may be an effective strategy to treat muscle fibrosis, and in a preliminary study, administration of miR-29 by adeno-associated virus (AAV) delivery resulted in a favorable anti-fibrotic response in *mdx* mice [65].

Another miR, miR146a-5p (miR146), which has been shown to inhibit renal fibrosis [66], has recently also been studied for its potential to reduce skeletal muscle fibrosis after injury. Sun et al. showed that miR146 levels were significantly reduced after a mouse model of acute contusion of the TA muscle [67]. In vitro, transfection of C2C12 myoblasts with miR146 led to reduced gene and protein expression of SMA, vimentin, and type I collagen. Transfection of miR146 in vivo after acute contusion also reduced the expression of these pro-fibrotic markers [67]. Furthermore, SMAD4 was shown to be a direct target of miR146, and overexpression of SMAD4 attenuated the anti-fibrotic effect of miR146 in vivo. This suggests that miR146 may attenuate skeletal muscle fibrosis by downregulation of SMAD4 [67].

## 6. TGF-β-Targeted Treatments of Skeletal Muscle Fibrosis

Based on the role of TGF-β in skeletal muscle fibrosis, modulation of TGF-β signaling is believed to be a useful target for developing therapies for skeletal muscle myopathies (Table 2). Inhibition of TGF-β expression by small interfering RNA (siRNA) and short hairpin RNA (shRNA) as well as knockdown of the TGF-β receptor has been studied extensively in the treatment of pulmonary and liver fibrosis, but less in the context of skeletal muscle fibrosis [68,69,70]. More recently, however, antisense oligonucleotides (AOs), which are synthetic, short single-stranded oligodeoxynucleotides that can alter RNA and modify protein expression, have also been studied as anti-fibrotic agents [71]. These polymers are believed to be more attractive due to the transience of target knockdown and the approval of two AO drugs for the treatment of genetic diseases, including DMD [72]. For example, in the context of skeletal muscle fibrosis, intramuscular injection of AOs for ALK-5 (the TGF-β type-I receptor) decreased the expression of pro-fibrotic genes in *mdx* mice [73].

Furthermore, suramin, a TGF-β1 receptor antagonist, has been widely studied for its anti-fibrotic properties [74]. In *mdx* mice, suramin has been shown to significantly decrease fibrosis and reduce muscle fiber damage in TA and biceps brachii muscle. This attenuation was also accompanied by a reduction in forelimb muscle strength impairment [75]. Decorin, another inhibitor of TGF-β signaling, has also been shown to reduce pathology and α-SMA-positive cells in Sharp-1^−/−^ mice [51].

Angiotensin-converting enzyme (ACE) inhibition has also been proposed as a therapeutic approach for fibrosis due to the aforementioned involvement of Ang-II and the AT-1R in the process. In one study, Morales et al. tested the effects of the ACE inhibitor enalapril on fibrosis in both sedentary and exercised (to exacerbate pathology and worsen disease progression) *mdx* mice [76]. Enalapril was shown to reduce skeletal muscle damage in both sedentary and exercised *mdx* mice as well as improve gastrocnemius muscle strength. This was accompanied by a reduction in levels of the ECM proteins collagen III and fibronectin in the gastrocnemius. Interestingly, however, TGF-β1 expression and signaling were not significantly reduced after enalapril treatment, but rather CTGF expression was indeed reduced by enalapril [76]. Burks et al. also studied the effect of ACE inhibition on muscle remodeling, although in sarcopenic mice using losartan, an Ang-II receptor antagonist [77]. Specifically, mice treated with losartan prior to cardiotoxin administration in the TA muscle exhibited significantly less fibrotic tissue and greater functional recovery than placebo-treated mice. In this study, losartan was shown to blunt TGF-β signaling compared to placebo [77]. 

Based on the involvement of ROS in the fibrotic process, the transcription factor NF-E2-related factor 2 (Nrf2), which protects against oxidative stress by inducing antioxidant enzymes such as NAD(P)H quinone oxidoreductase 1 (NQO1) through the antioxidant response element (ARE) has been studied in the fibrosis of different organs, including hepatic fibrosis and renal fibrosis [78,79]. With respect to skeletal muscle, Sun et al. studied the effect of Nrf2 activation by sulforaphane (SFN), an isothiocyanate found in cruciferous vegetables such as broccoli, in *mdx* mice [80]. The *mdx* mice treated with SFN demonstrated attenuated deposition of collagen in skeletal muscles, and levels of collagen I, fibronectin, and α-SMA were reduced [80]. SFN treatment also promoted ECM degradation by decreasing the inhibitors of TIMP-1 and plasminogen activator inhibitor-1 (PAI-1). In addition to the reduction in structural dystrophic features, SFN treatment also improved muscle function assessed by forelimb grip strength in *mdx* mice. These effects were likely mediated by Nrf2 activation, as SFN-treated mice exhibited increased expression of Nrf2 and the phase II detoxification enzymes heme oxygenase-1 (HO-1) and NQO1. SFN also strongly suppressed TGF-β1 expression and activation in *mdx* muscle, suggesting that SFN may lead to Nrf2-mediated suppression of TGF-β which attenuates muscle fibrosis [80]. Another compound, astaxanthin, has also been studied in a rat model of joint immobilization-induced muscle (soleus) fibrosis [81]. Astaxanthin, found mainly in marine organisms such as salmon, is an effective ROS scavenger with powerful antioxidant activity [82]. Following 14 days of immobilization, increased production of ROS and increased expression of TGF-β and α-SMA were observed, but these changes were attenuated by supplementation with100 mg/kg/day of astaxanthin [81].

## 7. Conclusions

Enhanced activity of the TGF-β pathway plays a critical role in the pathogenesis of many different myopathies. TGF-β1 levels have been shown to be elevated in plasma, serum, and muscle tissue of patients with different skeletal muscle myopathies. Through different mechanisms, overexpression of TGF-β1 leads to the formation of fibrotic tissue in a process of dysregulated muscle regeneration. In vitro experiments have shown that culturing cells with TGF-β1 results in decreased myogenic protein expression and increased fibrotic protein expression [41,83]. In vivo experiments have demonstrated that TGF-β1 treatment results in increased collagen content of muscle as well as reduced fiber CSA [42,84]. Scleraxis and Sharp-1 have been identified as potential transcription factors that are upregulated by TGF-β signaling that drive fibrosis [42,85]. Furthermore, fibrinogen and osteopontin have been identified as proteins that may enhance TGF-β expression and fibrosis by induction of an inflammatory cascade [53,55]. Finally, ROS, derived from Ang-II, or NOX, have been implicated as sources that increase TGF-β and collagen expression [57,58]. Potential promising fibrosis therapeutics targeting TGF-β signaling include TGF-β1 and antagonists, ACE inhibitors, antioxidants, and Nrf2 activators.

One point worthy of noting is the fact that most fibrotic skeletal muscles also demonstrate myofiber atrophy. For example, in addition to the extensive fibrosis associated with DMD, ALS, and PAD, muscles from patients with these conditions also demonstrate significant muscle atrophy. Muscle unloading as a result of tenotomy also results in significant muscle fiber atrophy as well as fibrosis [86]. Therefore, it is possible that there is an association between muscle fiber atrophy and fibrosis. As mentioned in this review, TGF-β1 has also been shown to significantly reduce myofiber size. Therefore, it is possible that resetting muscles to altered length (as a result of myofiber atrophy) may initiate the fibrotic process in an attempt to restore or fix the length [44]. Future research should determine the atrophy-dependent and independent effects of TGF-β on skeletal muscle fibrosis and clarify the relationship between atrophy and fibrosis.

The timeline of changes in TGF-β signaling in different myopathies, as well as in the normal process of regulated muscle regeneration, remains unknown. Therefore, future studies will need to explore the specific timeline of TGF-β dysregulation to establish optimal therapeutic intervention. Additionally, most of the TGF-β-targeted treatments of skeletal muscle fibrosis have only been studied in cell culture and in animal models, but very limited numbers of human studies exist. Therefore, future research should include clinical trials of the promising compounds to establish their safety and efficacy. It is also possible that a multi-targeted approach may be optimal, so future studies should investigate the effects of a combination of targets. Another important question that remains to be answered is whether alterations in TGF-β signaling are functionally linked to muscle performance and functional outcome in patients suffering from myopathies characterized by elevations in TGF-β. However, an important consideration when designing these studies is the dual role of TGF-β as pro-fibrotic but also anti-inflammatory. One possible way to address this is by regionally restricted administration of the treatment so that the function is limited to only skeletal muscle tissue. Repressing TGF-β signaling may have therapeutic potential, but it is first important to further understand the intracellular mechanism of the pathway to address potential off-target effects.

## Figures and Tables

**Table 1:** Transforming growth factor-beta (TGF-β) in fibrosis-associated skeletal muscle myopathies.

Study	Ref.	Myopathy	Source	Findings
Bernasconi et al., 1995	[14]	DMD	Muscle	Higher TGF-β1 gene expression in DMD vs controls TGF-β1 positively correlated with fibrosis
Ishitobi et al., 2000	[15]	DMD	Plasma	Higher TGF-β1 concentrations in DMD vs controls
Chen et al., 2005	[17]	DMD	Muscle	Strong induction of TGF-β pathway in symptomatic DMD patients vs controls by mRNA profiling
Song et al., 2017	[16]	DMD	Muscle	Higher TGF-β1 protein expression in DMD vs controls TGF-β1 positively correlated with degree of pathology and clinical severity
Ilzecka et al., 2002	[22]	ALS	Serum and CSF	Higher serum TGF-β1 concentrations in terminal status ALS patients vs controls CSF TGF-β1 positively correlated with duration of ALS
Si et al., 2015	[23]	ALS	Muscle	Higher TGF-β1 gene and protein expression in ALS patients vs controls TGF-β1 expression inversely correlated with muscle strength
Ha et al., 2016	[28]	PAD	Muscle	Higher TGF-β1 protein expression in PAD vs controls TGF-β1 expression increased with advancing disease stage TGF-β1 expression correlated with fibrosis
Matt et al., 2009	[30]	MFS	Serum	Higher TGF-β1 expression in patients with MFS vs controls

DMD: Duchenne muscular dystrophy; ALS: amyotrophic lateral sclerosis; PAD: peripheral artery disease; CSF: cerebrospinal fluid; MFS: Marfan syndrome.

**Table 2:** TGF-β-targeted treatments.

Study	Ref.	Compound	Model	Effect	Mechanism
Kemaladewi et al., 2014	[73]	ALK-5 AO	*mdx* mice	Decreased fibrotic gene expression	AO-mediated exon skipping
Taniguti et al., 2011	[75]	Suramin	*mdx* mice	Decreased fibrosis	TGF-β1 receptor antagonist
Acharjee et al., 2014	[51]	Decorin	Sharp-1^−/−^ mice	Reduced fibrotic pathology	Inhibitor of TGF-β signaling
Morales et al., 2013	[76]	Enalapril	*mdx* mice	Reduced ECM proteins	ACE inhibitor
Burks et al., 2011	[77]	Losartan	sarcopenic mice	Reduced fibrotic tissue following cardiotoxin administration	ACE inhibitor
Sun et al., 2016	[80]	Sulforaphane	*mdx* mice	Attenuated progression of fibrosis	Nrf2 activator
Maezawa et al., 2017	[81]	Astaxanthin	Rat ankle joint immobilization	Decreased fibrosis	Antioxidant/ROS scavenger

ALK-5: TGF-β type I receptor; AO: antisense oligonucleotide; ECM: extracellular matrix; ACE: angiotensin-converting enzyme; Nrf2: NF-E2-related factor 2.
